# Locus Ceruleus Norepinephrine Release: A Central Regulator of CNS Spatio-Temporal Activation?

**DOI:** 10.3389/fnsyn.2016.00025

**Published:** 2016-08-26

**Authors:** Marco Atzori, Roberto Cuevas-Olguin, Eric Esquivel-Rendon, Francisco Garcia-Oscos, Roberto C. Salgado-Delgado, Nadia Saderi, Marcela Miranda-Morales, Mario Treviño, Juan C. Pineda, Humberto Salgado

**Affiliations:** ^1^Neurobiology of Stress Laboratory, Facultad de Ciencias, Universidad Autónoma de San Luis PotosíSan Luis Potosí, Mexico; ^2^School for Behavior and Brain Sciences, University of Texas at DallasRichardson, TX, USA; ^3^Department of Psychiatry, University of Texas SouthwesternDallas, TX, USA; ^4^Laboratory of Cortical Plasticity and Learning, Universidad de GuadalajaraGuadalajara, Mexico; ^5^Electrophysiology Laboratory, Centro de Investigaciones Regionales “Dr. Hideyo Noguchi”, Universidad Autónoma de YucatánMérida, Mexico

**Keywords:** norepinephrine, adrenoceptors, stress, fight-or-flight response, ADHD, depression, psychosis, anxiety

## Abstract

Norepinephrine (NE) is synthesized in the Locus Coeruleus (LC) of the brainstem, from where it is released by axonal varicosities throughout the brain via volume transmission. A wealth of data from clinics and from animal models indicates that this catecholamine coordinates the activity of the central nervous system (CNS) and of the whole organism by modulating cell function in a vast number of brain areas in a coordinated manner. The ubiquity of NE receptors, the daunting number of cerebral areas regulated by the catecholamine, as well as the variety of cellular effects and of their timescales have contributed so far to defeat the attempts to integrate central adrenergic function into a unitary and coherent framework. Since three main families of NE receptors are represented—in order of decreasing affinity for the catecholamine—by: α_2_ adrenoceptors (α_2_Rs, high affinity), α_1_ adrenoceptors (α_1_Rs, intermediate affinity), and β adrenoceptors (βRs, low affinity), on a pharmacological basis, and on the ground of recent studies on cellular and systemic central noradrenergic effects, we propose that an increase in LC tonic activity promotes the emergence of four global states covering the whole spectrum of brain activation: (1) sleep: virtual absence of NE, (2) quiet wake: activation of α_2_Rs, (3) active wake/physiological stress: activation of α_2_- and α_1_-Rs, (4) distress: activation of α_2_-, α_1_-, and β-Rs. We postulate that excess intensity and/or duration of states (3) and (4) may lead to maladaptive plasticity, causing—in turn—a variety of neuropsychiatric illnesses including depression, schizophrenic psychoses, anxiety disorders, and attention deficit. The interplay between tonic and phasic LC activity identified in the LC in relationship with behavioral response is of critical importance in defining the short- and long-term biological mechanisms associated with the basic states postulated for the CNS. While the model has the potential to explain a large number of experimental and clinical findings, a major challenge will be to adapt this hypothesis to integrate the role of other neurotransmitters released during stress in a centralized fashion, like serotonin, acetylcholine, and histamine, as well as those released in a non-centralized fashion, like purines and cytokines.

***Berserk***

“*Early 19th century (originally as a noun denoting a wild Norse warrior who fought with frenzy): from Old Norse berserkr (noun), probably from birn-, bjorn (bear)* + *serkr “coat,” but also possibly from berr “bare” (i.e., without armor)*.” Oxford Dictionary.

“*His (Odin's) men rushed forwards without armor, were as mad as dogs or wolves, bit their shields, and were strong as bears or wild oxen, and killed people at a blow, but neither fire nor iron told upon them. This was called Berserkergang*.” Ynglinga saga and Laing Samuel (1889). The Heimskringla or the Sagas of the Norse Kings. London: John. C. Nimo. p. 276.

“*If a soldier survives the berserk state, it imparts emotional deadness and vulnerability to explosive rage to his psychology and permanent hyperarousal to his physiology—hallmarks of post-traumatic stress disorder in combat veterans. My clinical experience with Vietnam combat veterans prompts me to place the berserk state at the heart of their most severe psychological and psycho-physiological injuries*.” Shay Jonathan (1994). Achilles in Vietnam. New York: Scribner. p. 98. ISBN 0-689-12182-2.

## Introduction

### Neurotransmitters controlling the spatio-temporal brain activation patterns

Evolution has shaped the mammalian brain during millions of years, endowing it with redundant and inter-related neurotransmitter networks to manage and administer stress. The characteristics of the “*berserk*,” the ultimate warrior—superhuman physical strength, insensitivity to pain, lack of concern for the consequences of his actions—are possibly the display of an extreme state, an upper limit of human physical and mental condition at the core of norepinephrine (NE)-induced states.

**Figure 1:**
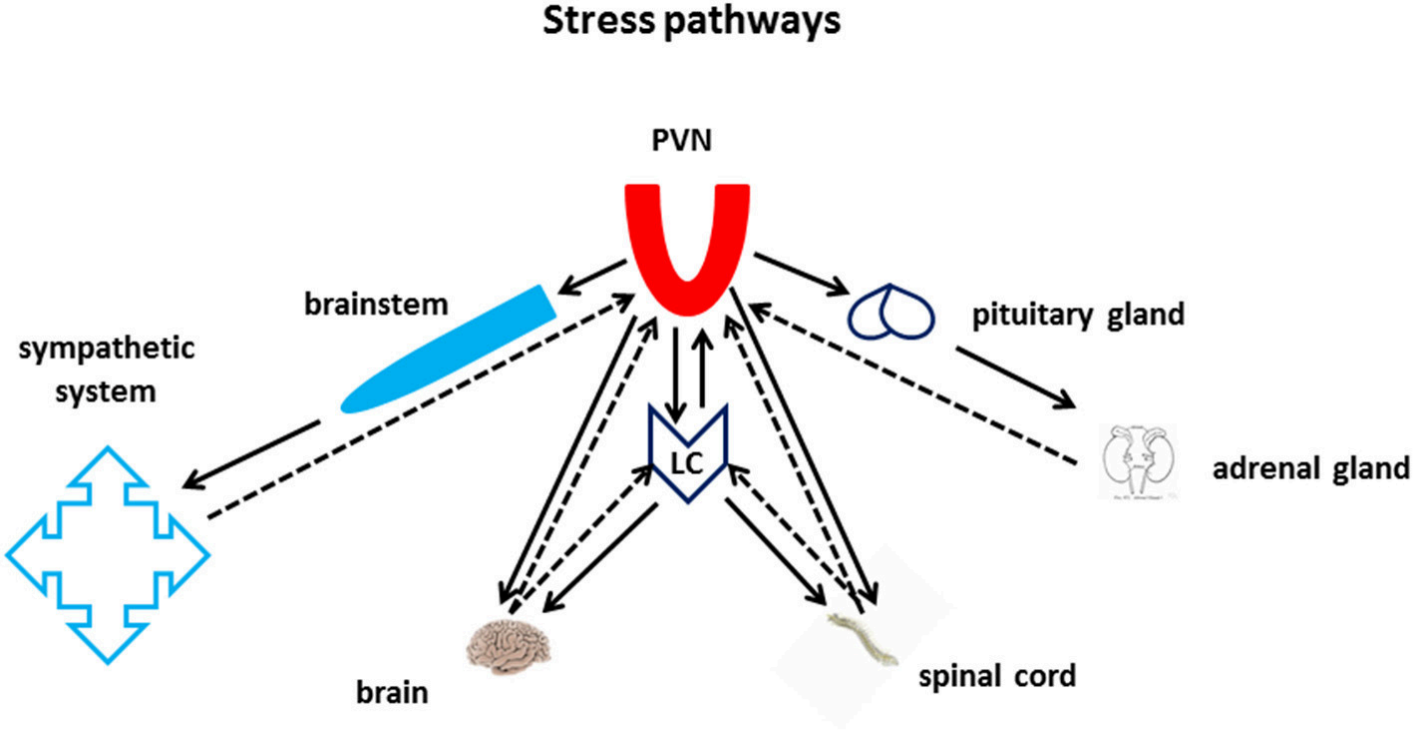
**Stress pathways**. The Nucleus Paraventricularis of the Hypothalamus (PVN) and the Locus Ceruleus lie at the core of the CNS stress pathways. Both areas are at the center of an intense bi-directional information exchange with multiple targets in the periphery, within the brain, and with each other. The PVN sends and receives information to and from the autonomic nervous system through the brain stem, and from and to the periphery through the neuroendocrine axes. The LC sends and receives information to and from the spinal cord and the whole brain. Furthermore, PVN and LC also share monosynaptic bi-directional communication through the medial forebrain bundle.

Although a variety of hormones may turn on neuronal circuits for the execution of energetically demanding behavioral tasks, only a fistful of neurotransmitters have the capability to actually regulate the global state of activation of the *whole* brain, managing effectively and parsimoniously the necessarily limited energy/power capability of the brain and of the whole organism. The NE-releasing Locus Ceruleus (LC) is anatomically and functionally intertwined with the brain area which is arguably the major recipient of stress-related information: the paraventricular nucleus of the hypothalamus (PVN, Figure 1). Other hypothalamic nuclei also impinge upon the LC. Among them the hypocretin-expressing nuclei in the lateral hypothalamus (Henny et al., 2010; Carter et al., 2012). The hypothalamus-LC axis controls input and output information from and to the autonomic system through the brainstem, to and from the neuroendocrine system through the pituitary gland and numerous gland-to-brain biochemical feedback loops, as well as all the rest of the central nervous system (CNS), through brain and spinal cord volume transmission (Figure 1).

Further hints of the biological importance and pervasiveness of central adrenergic function come from the analgesic properties of NE and its agonists (Simpson and Lin, 2007), by its important role in the control of body temperature, like during inflammatory response (Bencsics et al., 1995; Ordway et al., 2007; Osaka, 2009), as well as from the observation that other relevant input to the LC originate from nuclei coordinating vital functions like sex/reproduction (Nucleus Paragigantocellularis), respiration (Parabrachial and Solitary Tract Nuclei), and vestibular balance control (Simpson and Lin, 2007).

The control of the above functions is likely retained in the evolution from lower to higher mammals (Tohyama et al., 1974), but the increase in brain size and complexity associated with its disproportionate anatomical development makes the mammalian CNS particularly vulnerable to sudden surges in energy consumption caused by stressful situations. This latter evolutionary purpose might have further strengthened the importance of LC as master energy hub (Berridge and Waterhouse, 2003; O'Donnell et al., 2012), enhancing—particularly in humans—its role in the etiology of stress-related conditions.

### Functional and anatomical peculiarity of the LC/NE system

Many hormones have a potential for global control of energy expenditure and activity regulation. Among them—for instance—the corticosteroid system is well placed for exerting a global and sophisticated biochemical regulation of energy demand and distribution (De Kloet, 2004), but lacks the property of anatomical and functional contiguity that the LC possesses. For similar reasons, the cytokine network, which also has the potential to control the brain (and bodily) global energy distribution (Guijarro et al., 2006), also does not seem to qualify as “central energy master.”

Central cholinergic fibers made up a highly divergent and almost ubiquitous release system (McKinney and Jacksonville, 2005; Smythies, 2005a). However, the existence of a large number of nuclei and brain areas that *independently* control the release of acetylcholine (Ach; Nucleus Basalis of Meynert, medial septum, latero-dorsal tegmentum, etc.), each toward or within their respective anatomical targets suggests that the cholinergic system hardly exerts a genuinely *centralized* control of energy expenditure.

The central histaminergic system stemming from the tuberomammillary nucleus of the hypothalamus appears to play a powerful and genuinely centralized role in triggering an emergency and alert maintenance response (Wada et al., 1991; Sakata et al., 1997; Shan et al., 2015). To our present knowledge, though, the histaminergic system does not appear to display a repertoire of cellular and synaptic actions paralleling the complexity and flexibility of the LC/adrenergic system, which is perhaps rivaled in its pervasiveness and variety of effects only by the Raphe/5HT system (Heisler et al., 2003; Zhou et al., 2005). In this respect, only serotonergic projections from the Raphe nucleus and the cholinergic fibers from the basal forebrain (Nucleus Basalis of Meynert) reach the extent and density of LC adrenergic projections throughout the CNS (Smythies, 2005b). The importance of the serotoninergic system in the coordination of the stress response has been reviewed elsewhere (Waselus et al., 2011).

While we highlight the importance of developing a comprehensive theory integrating the roles of the many neurotransmitters involved in the stress response, we will henceforth limit our discussion on the role of NE. In the following sections we will discuss experimental evidence relating central NE function to activities related to stress (stress perception, elaboration, and execution of a stress-ridding plan, as well as storage—or deletion—of related memories), and will make an attempt to integrate previous literature into a qualitative model in which increasing levels of NE co-ordinate the activity of different brain areas, inducing global brain states with increasing energy consumption and stress levels. We will only briefly mention the effects of NE on long-term processes, which we have recently reviewed elsewhere (Salgado et al., 2016).

It is worthwhile emphasizing the genuinely global nature of NE, differing from its chemical precursor dopamine, whose cortical projecting axons target more selectively the prefrontal cortex (Robbins and Arnsten, 2009). For the sake of clarity, we would like to highlight that stress activates two distinct pools of NE: a central one and a peripheral one, the latter associated with sympathetic nervous system activation. While the interaction between the two pools is essential to the understanding of the systemic effects of stress, only the former will be considered in the present discussion.

### Stress, HPA axis, and LC activation

For the purpose of this discussion, we will broadly define *stress* as any situation in which an organism *increases* its energy consumption *beyond an expected or biologically bearable range* (which greatly varies even among individuals of the same species), and as *stressor* its objective or perceived source. We will get back to this definition of stress in Section LC-CNS Interactions. In the presence of most types of stressors the brain carries out the following (conscious or subconscious) functions: (1) evaluation of the stressor characteristics (short- or long-term intensity, duration, and consequences), (2) elaboration of a strategy to eliminate the stress(or), (3) execution of such plan, and (4) long-term storage (or in some case erasing) of stressor-related memories. Among these memories are the inner representations of the stress as a measure of potential danger, as well as the representation of one or more actual stress exit strategies, and their perceived effectiveness (or lack thereof).

In the historical context of the studies of the stress response, a critical element of adrenergic effects had been recognized early in the interaction between the hypothalamus-pituitary-adrenal gland (HPA) neuroendocrine axis and LC reviewed in Gold and Chrousos (2002) and Gold (2015). In fact, NE-releasing neurons of the LC are important targets of corticotropin-releasing hormone (CRH)-producing hypothalamic neurons from the PVN (Nicolaides et al., 2015), as well as from other limbic areas including the amygdala (Ordway et al., 2007), whose activity in turn stimulates LC leading to NE release. The importance of the PVN-LC axis is underscored by the observation that the inactivation of glucocorticoid receptors in the LC induces depression-like symptoms in a mouse model (Chmielarz et al., 2013).

**Figure 2:**
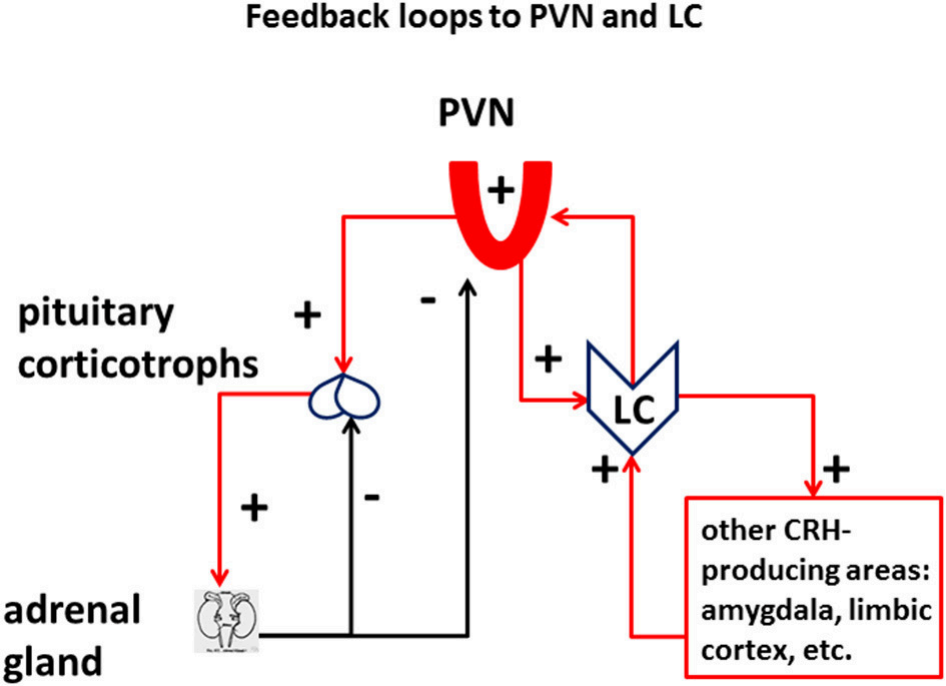
**Feedback loops to PVN and LC: Vulnerability of the LC in the stress axes**. The LC is integral part of the stress response, in addition to the HPA axis. Different from the HPA axis, which receives a double negative-feedback (minus signs) of corticosteroids from the suprarenal gland, both at the level of the pituitary and the paraventricular nucleus of the hypothalamus (PVN), PVN, and other CRH-releasing cells in the CNS are connected to the LC through a positive-feedback loop (plus signs), which has the potential to derange the energy equilibrium of the system.

A critical feature of the well-studied HPA response to stress is the negative feedback between the production of glucocorticoids and the activation of the HPA axis, which occurs both at the level of CRH-producing neurons in the PVN of the hypothalamus as well as in pituitary ACTH-producing corticotrophs (Figure 2). An energetically meaningful consequence of the elevation of glucocorticoid levels is the parallel shut-down or at least decrease of the high-energy consuming immune adaptive system, which in turn may increase the chance of infection and cancer in chronically stressed individuals (Reiche et al., 2004).

Importantly—unlike the glucocorticoid negative-feedback on HPA axis—the activation of the PVN/CRH/NE/LC branch of the stress response not only does *not* produce a negative feedback (Gold, 2015), but produces a *positive* feedback which opposes and jeopardizes the closure of the HPA loop associated with glucocorticoids (Figure 2). The presence of a feedforward loop between CRH-producing areas of the cortex and of the hypothalamus and the LC is a risk factor in the induction of maladaptive plasticity of the stress system, which greatly enhances its vulnerability to intense and/or chronic challenge. Systemic inflammation can be considered as the opposite phenomenon, whereby a combined action of pro-inflammatory cytokines induces a temporary state of physical apathy and inaction (Haroon et al., 2012; Miller et al., 2013). A consequence of such “*sick response*” is to spare systemic energy and promote a prompt recovery of the organism affected by a viral or bacterial infection.

**Figure 3:**
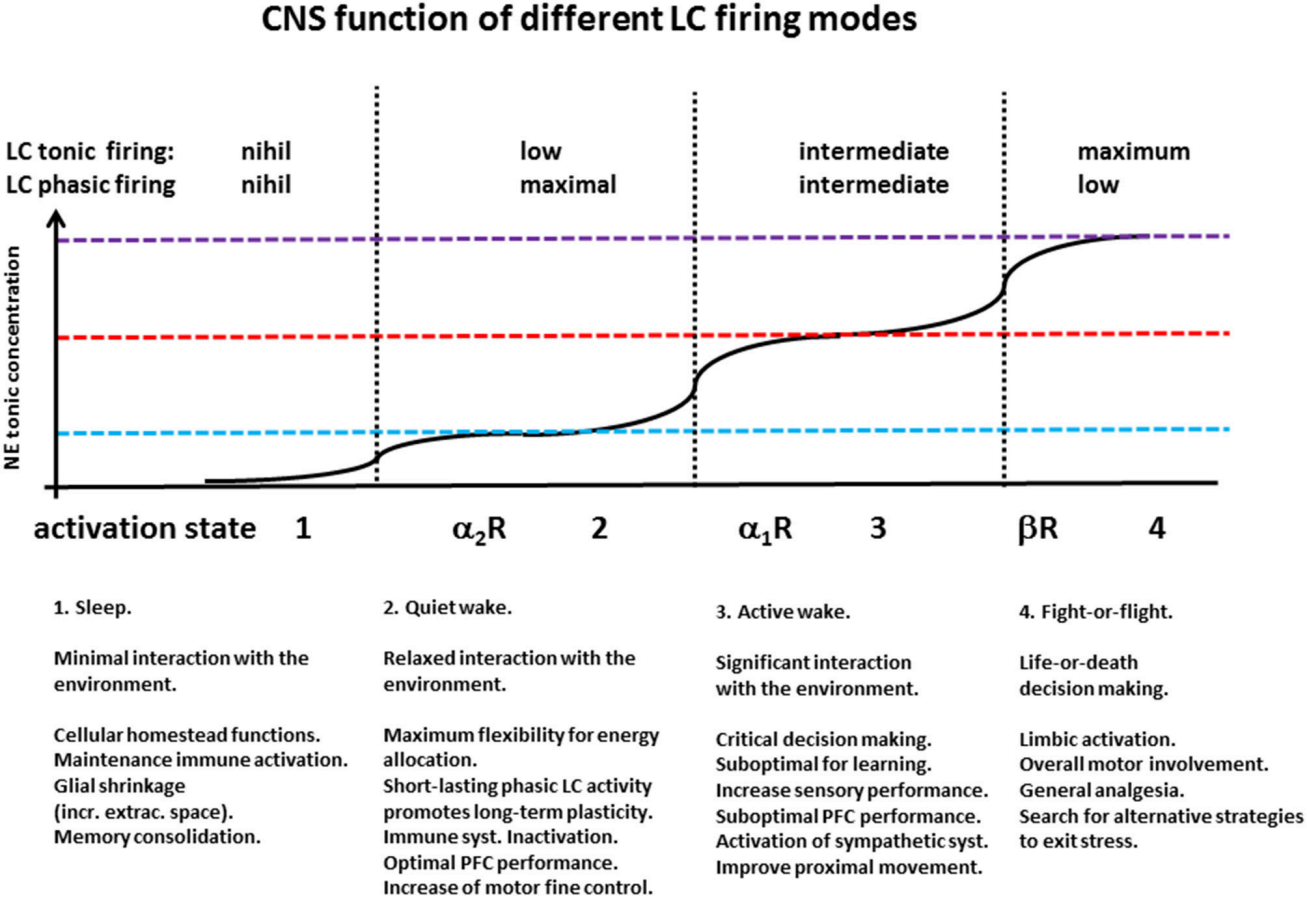
**CNS function of different LC firing modes**. Seminal work from Aston-Jones groups has shown the existence of a relationship between behavioral states and LC tonic and phasic firing patterns: During sleep, LC cells display low or no activity (vertical axis in arbitrary units—A.U.); during quiet wake they display modest tonic firing, and phasic responses to behavioral stimuli; in conditions of intermediate tonic release, associated with moderate stress and energy demand, LC presents its highest phasic response during biologically relevant behavioral responses; the highest level of LC tonic firing occurs in situations of arousal and fight-or-flight response, and is associated with the lowest levels of phasic LC activity.

Decade-long seminal work from the group of Aston-Jones and Waterhouse provides a solid ground for assessing the basic functions and activity dynamics of the LC (Rajkowski et al., 1994; Aston-Jones et al., 1998; Usher et al., 1999; Aston-Jones and Cohen, 2005; Aston-Jones and Waterhouse, 2016). Using mostly *in vivo* electrophysiological recordings from both primate and rodent models, this body of work has shown that LC displays virtually no activity during the sleep phase, whereas during the wake state it displays two emergent firing patterns: a tonic one, associated with the arousal level of the animal, and a phasic one, related with decision making and attention. Importantly, the extent of LC phasic firing appears to follow an inverse-U shape function with respect to the levels of tonic LC firing. In fact, while *low* levels of tonic firing—as during low arousal level—are insufficient to elicit a consistent behavioral response, *intermediate* tonic levels yield optimal phasic firing, whereas *high* levels of tonic firing—associated with high arousal and limbic activation—yield low phasic response as LC units firing activity gets closer to saturation. The eventual overall effect of NE depends on the spatio-temporal pattern of its volume release (Fuxe et al., 2010), on the nature of the cellular and synaptic targets affected, and on the type of receptor activated (summarized in Figure 3).

### Molecular, cellular, and synaptic effects of NE

NE is released in the brain by axonal varicosities via volume transmission (Grzanna, 1980; Jones and Yang, 1985; Agnati et al., 1995). NE receptors have been first studied in the periphery and subsequently identified throughout the whole CNS, with different densities and regional specializations as reviewed earlier (Ramos and Arnsten, 2007). In terms of molecular effects, they can be categorized into three main groups, in descending order of affinity: α_2_Rs, (≈50 nM), α_1_Rs (≈300 nM), and βRs (≈0.7–0.8 μM) reviewed in Ramos and Arnsten (2007). Both α_2_- and β-Rs are known to activate guanosine-dependent (G–)protein receptors, each affecting adenylyl cyclase in opposite directions, namely by decreasing (α_2_Rs) or increasing (βRs) the intracellular concentrations of cyclic adenosine monophosphate (cAMP). In contrast, α_1_Rs activate phospholipase C (PLC), thus triggering the synthesis of intracellular diacylglycerol and activation of protein kinase C as well as of inositol phosphate, which in turn releases Ca^2+^ from intracellular stores (Ramos and Arnsten, 2007).

**Figure 4:**
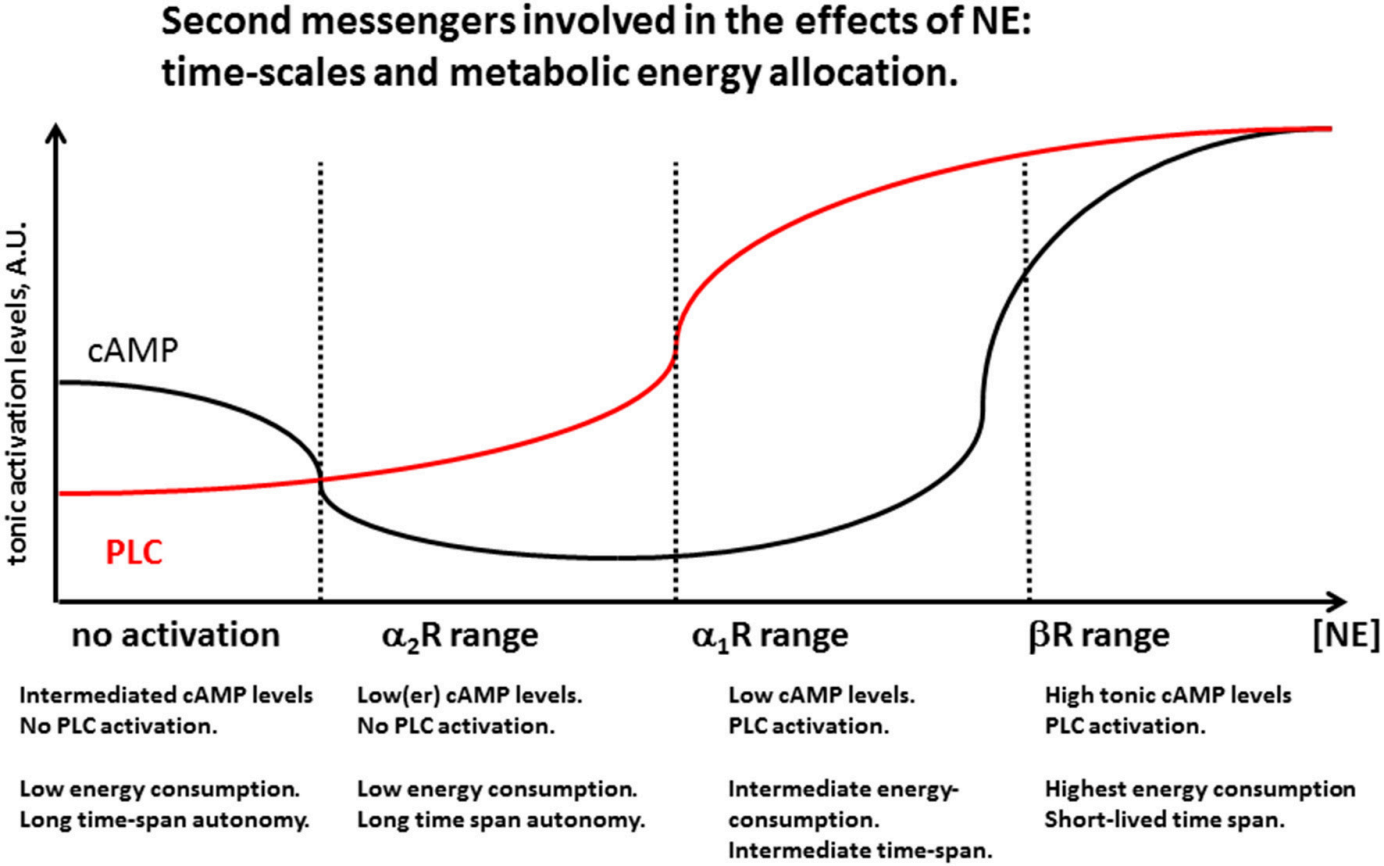
**Second messengers involved in the effects of NE: time-scales and metabolic energy allocation**. Increasing levels of NE activate noradrenergic receptors by first decreasing cAMP levels by activation of α2Rs, probably reducing homestead maintenance cellular function active during sleep. A further increase in NE concentration activates α1R, activating the phospholipase C (PLC) cascade while cAMP levels are still low. For still higher levels both PLC and cAMP levels are heightened, consistent with highest levels of cellular activation. Energetic considerations suggest that this high-demand state need to be associated with decreased function in at least some brain areas, and has necessarily to be short-lasting, in order to prevent depletion of organismic energy stores and desensitization of membrane receptor mechanisms. Periods of brief and intense LC activation like during its phasic release may induce temporary activation of βRs associated with memory and learning. Prolonged high LC activity may be detrimental for learning and memory as it would necessarily reduce phasic LC activity and reduce the spatial and temporal specificity of βR synaptic effects.

The existence of widespread families of high-affinity neurotransmitter receptors (NE α_2_Rs, M_2_ muscarinic, D_2_ dopaminergic) whose activation decreases adenylyl cyclase activity (G_i_) suggests that basal (tonic) cytosolic levels of cAMP are not zero, and that they concur to the regular maintenance cellular processes active during cell rest. As a corollary, we hypothesize that the inactivity of the CNS noradrenergic system—similar to that of the other monoaminergic and the cholinergic system—during sleep is associated with a non-zero level of cAMP (Figure 4), and a tonic level of cellular energy expenditure. Such ground-level of cellular metabolic activity is possibly necessary to carry out a number of functions including a temporary enhancement of immune function during the resting phase (Kamath et al., 2015) and memory consolidation (Wilson and McNaughton, 1994; Barnes and Wilson, 2014; Figure 3). A slight increase in the concentration of neurotransmitters activating a G_i_ (in the case of NE up to 100 nM) could be sufficient to shut down such sleep-associated maintenance cellular processes and re-direct cellular metabolic energy to the quiet-wake related activities. Only relatively higher NE concentrations (around or above 0.4 μM) would be able to solidly activate the PLC cascade and increase cAMP levels above its resting levels (Figure 3), increasing the cellular supply for more energetically demanding biological activities (Figure 4).

Cellular and synaptic adrenergic modulation (Salgado et al., 2016) suggests that the activation of the cortical branch of the LC/adrenergic system could simultaneously perform two tasks: (1) single neuron activation by modulation of intrinsic conductances, with consequent local mobilization of large amounts of metabolic energy, and (2) temporary shut down or depression of the activity of other cortical areas that are unnecessary or irrelevant to the resolution of a particular behavioral contingence. This end could be achieved by a combination of selective depression of excitatory and enhancement of inhibitory synaptic transmission (Waterhouse et al., 1991; Sessler et al., 1995; Salgado et al., 2016).

Heterogeneous mechanisms of adrenergic modulation in different cortical areas like sensory vs. prefrontal cortices (Salgado et al., 2011, 2012b; Roychowdhury et al., 2014) together with activity-dependent modulation may concur to a functional selective enhancement or depression of neuronal activity in specific areas (Hains and Arnsten, 2008; Arnsten et al., 2012; Edeline, 2012; Roychowdhury et al., 2014). In the following sections we will review experimental evidence of effects of the noradrenergic receptor families mentioned above, from clinics and animal models, in an attempt to condense the related information in an integrated view. In many cases it will be hard to guess how a particular cellular or synaptic phenomenon participates into a behavioral function. In the tables we will either report the author's interpretation of their experimental finding, or will formulate a plausible one, keeping in mind that the same cellular experimental data may play different roles in a systemic function.

## α_2_Rs central modulation

α_2_Rs are present in many brain areas in both pre- and post-synaptic membranes, as well as in glia (Lee et al., 1998).

### Alertness and anxiolytic effects

Based on pharmacological observations in clinics and in animal models, the activation of α_2_Rs is deemed necessary for optimal performance in working memory and other tasks carried out with a strong prefrontal cortex (PFC) component (Gamo and Arnsten, 2011; Arnsten and Jin, 2012, 2014). Along with promoting working memory—and possibly related to it—α_2_Rs also appear to contribute to a plethora of functions such as attention and impulse inhibition (Brennan and Arnsten, 2008; Robbins and Arnsten, 2009). The activation of α_2_Rs decreases the inhibitory synaptic drive onto the tuberomammillary nucleus of the hypothalamus, contributing to alertness (Nakamura et al., 2013). α_2_R activation in the medial septum and hippocampus increases theta rhythm (Kitchigina et al., 2003), presumably enhancing cognition. The increase in spontaneous inhibitory postsynaptic currents (sIPSCs) in the PVN following α_2_R agonist application (Chong et al., 2004), together with the α_2_R-induced decrease in glutamatergic drive onto the ventral tegmental area (VTA) may contribute at least part to α_2_Rs anxiolytic properties.

### PFC activity modulation

Ample evidence exists that α_2_Rs directly modulate PFC activity (Kovács and Hernádi, 2003; Wang et al., 2010), reviewed in Arnsten and Li (2005). Most of this literature indicates a beneficial effect of α_2_R activation for working memory, although in some studies beneficial effects of α_2_R *blockers* have been described (Brown et al., 2012; Bari and Robbins, 2013). The α_2_R-induced block of N-methyl-D aspartate receptor (NMDAR)-mediated current (Liu et al., 2006), would corroborate a role in PFC learning for this receptor, but it could also represent a faster “clearance” of PFC reverberant circuits. Particularly remarkable is the evidence from multiple studies, of the beneficial effects of α_2_R-induced block of dendritic hyperpolarization-activated cyclic nucleotide (HCN) channels (Wang et al., 2011; Zhang Z. et al., 2013), whose age-related decline is considered an important cause of cognitive deterioration (Wang et al., 2011).

### Motor and sensory activity

α_2_R activation does modulate motor activity (Villégier et al., 2003), although not always in the same direction (Cathala et al., 2002; Carey and Regehr, 2009). α_2_R activation appears to modulate cerebellar activity, necessary for fine timing and control of distal movement (Hirono and Obata, 2006; Di Mauro et al., 2013; Lippiello et al., 2015). Little evidence is reported of α_2_R modulation of sensory areas, mainly in auditory (Leão and Von Gersdorff, 2002; Salgado et al., 2011) and olfactory (Nai et al., 2009) areas.

### Clinical and pre-clinical studies

Different—sometimes contradictory—evidence about the global effects elicited by α_2_R ligands may perhaps be explained as the result of two contrasting actions on *excitatory* (pro-convulsive) *presynaptic* α_2_Rs and *inhibitory* (anti-convulsive) *postsynaptic* α_2_Rs, as revealed by an epilepsy study (Szot et al., 2004). It is worth mentioning that the use of tricyclic medication, used as antidepressant, may induce α_2_R internalization (Cottingham et al., 2015), perhaps indicating that depression may be associated with or even caused by an increase in α_2_R expression, possibly elicited by a high NE tone associated with prolonged stress.

Overall, the activation of α_2_Rs by an increased but moderate NE tone (possibly ≤ 100 nM), appears to increase alertness, improve working memory, attention, PFC function in general, and enhance fine motor control and sensory processing, possibly acting on pre- and post-synaptic receptors carrying out opposite functions. Table 1 reports a series of α_2_R-mediated effects grouped per putative function.

**Table 1:** **Central effects of α_2_ adrenergic receptors**.

**Measured or putative function of α_2_R activation**	**Brain area**	**α_2_R-related physiological effect/finding**	**References**
**INCREASE IN AROUSAL AND GENERAL ACTIVITY**			
Modulation of NE release	LC	α_2_R are present pre- and post-synaptically and in glia	Lee et al., 1998
Increase arousal	Tuberomammillary nucleus	α_2_R activation decreases GABAergic synaptic transmission	Nakamura et al., 2013
Increase arousal and cognitive functions	Medial septum and hippocampus	α_2_R activation increase theta rhythm frequency	Kitchigina et al., 2003
Reduced stress response	PVN hypothalamus	α_2_R activation increases sIPSC frequency	Chong et al., 2004
Decreases limbic axis activation	VTA	α_2_R act decrease glutamatergic drive onto VTA cells	Jiménez-Rivera et al., 2012
Emotional memory consolidation during sleep	Human amygdala and hippocampus	α_2_R activation facilitates consolidation of memories	Groch et al., 2011
**PREFRONTAL CORTEX/WORKING MEMORY/EXECUTIVE FUNCTION**			
Improve Executive function	Systemic, in rodent, and primates	α_2_R activation	Arnsten and Li, 2005
Promote working memory	dlPFC	α_2_R activation promotes persistent firing	Arnsten, 2011
Modulates error detection	mPFC	LC lesion increases mPFC firing	Wang et al., 2010
		α_2_R activation decreases mPFC firing	
Promote working memory	PFC	α2 block decrease firing frequency (α2 activation increase firing frequency)	Kovács and Hernádi, 2003
Promote working memory	PFC	α_2_Rs block HCN channels	Wang et al., 2011
Promote working memory	PFC	α_2_Rs prolong persistent activity (up-states) through block of HCN channels	Zhang Z. et al., 2013
Modulate working memory	mPFC *in vivo*	α_2_R act decrease glutamatergic transmission fEPSP. Mixed effect on synaptic transmission on multi- unit population (could be due to effects on inhibitory transmission)	Ji et al., 2008
Improvement of working memory	PFC	α_2_R activation blocks HCN channels and increases excitability	Carr et al., 2007
		Promotes temporal summation	
Working memory	systemic	Block of α_2_R improves sustained attention and response inhibition	Bari and Robbins, 2013
Modulation working memory	PFC	α_2_R activation decrease NMDA currents	Liu et al., 2006
Increase false alarm/lower threshold for event detection	systemic	α_2_R activation increases false alarm	Brown et al., 2012
**MODULATION OF MOVEMENT CONTROL BY α_2_ ADRENOCEPTORS**			
Increase in locomotor activity	Systemic/overall brain	α_2_R agonists increase locomotor activity	Villégier et al., 2003
Decrease dopamine release/motor drive	Substatia Nigra pars compacta	α_2_R agonists activate a cationic current increasing sIPSC frequency	Cathala et al., 2002
Decrease motor learning	cerebellum	α_2_R activation decreases associative plasticity	Carey and Regehr, 2009
Promote fine movement control	cerebellum	α_2_R activation reduces IPSC	Hirono and Obata, 2006
Modulate cerebellar input	Cerebellar Purkinje cells	α_2_R activation reduce EPSC	Lippiello et al., 2015
Movement control fine tuning	Cerebellum	α_2_R activation increases and decreases GABA in different subareas	Di Mauro et al., 2013
**MODULATION OF SENSORY ACTIVITY BY α_2_Rs**			
Promotes olfaction	Olfactory bulb	α_2_R activation increases olfactory discrimination	Nai et al., 2009
Modulation of auditory sensitivity	Calyx of held	α_2_Rs activation decreases glutamatergic signaling but increases firing frequency	Leão and Von Gersdorff, 2002
Decrease auditory sensitivity	Auditory cortex	α_2_Rs activation increases GABAergic signaling	Salgado et al., 2011
**ROLE OF α_2_Rs BRAIN PATHOLOGY**			
Pro- and anti-convulsant effect	systemic	α_2_A *presynaptic* autoreceptors are responsible for the proconvulsant effect of α_2_R agonists	Szot et al., 2004
		α_2_ *postsynaptic* receptors are responsible for the anticonvulsant effect of α_2_R agonists	
Antidepressant effect	Systemic	Tricyclics induce β arrestin-mediated internalization of α_2_Rs	Cottingham et al., 2015
Antidepressant effect	mPFC	α_2_R activation reduces AMPAR currents	Yuen et al., 2014
Improve executive functions	mPFC	Cannabinoid receptors (which impair working memory) decrease α_2_R function	Cathel et al., 2014
Decrease distress in drug addiction (seeking) behavior	BNST	α_2_R activation decreases excitatory transmission	Egli et al., 2005
Intra-BNST α_2_R agonists inhibit drug seeking			

## α_1_Rs central modulation

A somehow controversial picture emerges from the literature concerning the roles of α_1_Rs, reporting either facilitatory or detrimental effects of cognitive function following the activation of α_1_Rs, depending on the assay used. Remarkable information comes from studies of different subtypes of α_1_R in constitutively activated mutant (CAM) mice, reviewed elsewhere recently (Nalepa et al., 2013). These studies suggest complex—sometime opposite—interplay of the different subtypes of α_1_Rs. CAM mice overexpressing α_1B_Rs display neurodegeneration and *grand mal*-like seizures, probably caused by an imbalance between excitatory and inhibitory synaptic currents. Behavioral assays on these animals suggest a role for α_1B_Rs in memory consolidation and fear-driven exploratory behavior (Knauber and Müller, 2000). On the other hand, α_1A_Rs CAMs live 10% longer than controls, and display improved memory and learning (Doze et al., 2011), opposite to α_1B_Rs CAMs (Collette et al., 2014). Another subtype of α_1_Rs, the α_1D_R appears to be inversely related to motor control, as α_1D_R KO mice perform better in the rotarod test (Mishima et al., 2004).

### General activation and emotion regulation

In general, α_1_Rs activation promotes wake and activity by directly affecting neurons (Schmeichel and Berridge, 2013; Igata et al., 2014), and possibly also by activating astrocytes (Pankratov and Lalo, 2015). Activation of α_1_Rs also concurs to the anorexigenic effect of NE and amphetamines (da Silva et al., 2014).

A specific and consequential effect of α_1_R activation is emotion control. The decrease of the inhibitory drive onto the VTA may indicate an increase in motivation (Velásquez-Martínez et al., 2015). α_1_Rs are also clearly involved in the stress response, as revealed by acute restraint stress (Alves et al., 2014), predatory stress (Rajbhandari et al., 2015), and maternal separation (Coccurello et al., 2014) studies. In agreement with its role in the stress response, block of α_1_Rs impairs HPA activation (Yang et al., 2012).

### Working memory and motor control

α_1_R positive modulation of working memory and other PFC function also seems to be solidly established by a wealth of data. For instance, α_1_R activation improves working memory deficit induced by applications of the GABA_A_R agonist muscimol (Hvoslef-Eide et al., 2015), while α_1_R block disrupts the “*go*” performance in a “*go-no-go*” task (Bari and Robbins, 2013). At the synaptic basis of these effects could lay an enhancement in glutamatergic function (Luo et al., 2014b, 2015a), which may, in turn, yield a general increase in firing frequency in the PFC (Zhang Z. et al., 2013). Other cellular and synaptic effects of α_1_R activation in the PFC, like an increase in inhibitory drive (Luo et al., 2015b) or a specific decrease in NMDAR-mediated response are not necessarily prone to similarly straightforward interpretations.

Motor effects of α_1_R activation appear to be associated with a generalized increase in motor activity (Villégier et al., 2003), accompanied by a reduced fine motor control (Aono et al., 2015), suggested also by improved rotarod performance of KO α_1_R mice (Mishima et al., 2004). A decreased glutamatergic cerebellar drive may concur to a reduced distal motor control (Lippiello et al., 2015).

### Sensory modulation, memory, and learning

Even more puzzling are the effects of α_1_R activation on sensory activity. While the effectiveness of α_1_R activation on sensory areas appears to be well established, its overall function remains enigmatic, possibly due to opposite effects on excitatory and inhibitory synaptic systems, as well as to a genuine heterogeneity of the response to different sensory modalities. For instance, α_1_R activation increases firing in the somatosensory cortex (Devilbiss and Waterhouse, 2000), but decreases firing frequency and responses to glutamate in the visual (Terakado, 2014) and in the auditory cortex (Manunta and Edeline, 1997; Dinh et al., 2009). In the latter—in turn—activation of α_1_Rs elicits opposite responses on electrically-evoked GABAergic transmission originating from different cortical layers (Salgado et al., 2011, 2012a). αRs (possibly α_1_Rs) are involved in auditory cortex activity-dependent plasticity evoked by electric or optogenetic stimulation of LC (Martins and Froemke, 2015).

The olfactory bulb is also not exempt from displaying apparently contrasting α_1_R-induced effects, like an increase of GABAergic response (Zimnik et al., 2013) and membrane depolarization (Nai et al., 2009). The effects of α_1_R activation on sensory areas may be related to sensory modality selection after adrenergic activation, and/or maintenance of the excitatory/inhibitory balance following intense activation.

On the other hand, α_1_R activation elicits clearly positive effects on memory and learning (Doze et al., 2011), as corroborated by studies on constitutively active α_1A_R mentioned earlier (Collette et al., 2014), and by a worsened learning and working-memory related performance in KO α_1_R mice (Spreng et al., 2001). An increase in rebound excitation and neuronal ensemble synchronization associated with an α_1_R–mediated increase in GABA release in the entorhinal cortex (Lei et al., 2007; Cilz et al., 2014) may be at the root of at least some of the α_1_R–induced improvements in learning and memory.

### Clinical data

Depression, psychosis, and numerous treatments for stress-related neuropsychiatric disease appear to modulate importantly α_1_Rs expression and function, although the direction of such modulation is not always consistent with illness or therapeutic effects. For instance, long-term administration of imipramine or electroconvulsive therapy increase the expression of α_1_Rs (Nalepa et al., 2002), but the antidepressant effects of other tricyclic antidepressants (TCAs) (Ramakrishna and Subhash, 2012) or quetiapine (Nikiforuk, 2013) reduce α_1_Rs expression. The interpretation of these results is further complicated by the age-dependence of α_1_Rs function (Deupree et al., 2007). Table 2 summarizes some of the systemic and cellular effects associated with α_1_Rs activation.

**Table 2:** **Central effects of α_1_ adrenergic receptors**.

**Measured or putative function of α_1_R activation**	**Brain area**	**α_1_R-related physiological effect**	**References**
**GENERAL ACTIVATION/METABOLISM**			
Wake promoting	Preoptic area hypothalamus, medial septum	α_1_R (and βR) activation promotes wake	Schmeichel and Berridge, 2013
General activation	Overall brain, astrocytes	α_1_R induces Ca-waves, ATP release in astrocytes	Pankratov and Lalo, 2015
Hyperexcitability	LC	Persistent α_1_R activation increases hyperexcitability	Igata et al., 2014
Brain activation	Brain, systemic	α_1_R activation induces Ca-waves	Ding et al., 2013
Food intake	Medial raphe	α_1_ block induces food intake	da Silva et al., 2014
**EMOTION/STRESS/MOOD/MOTIVATION**			
Promotes motivation	VTA	α_1_R activation decreases GABAergic IPSC	Velásquez-Martínez et al., 2015
Promotes emotional response	Insular cortex	α_1_R (and α_2_R) activation induce systemic response to acute restraint stress	Alves et al., 2014
Postnatal stress increase α_1_R sensitivity (fear)	Amygdala	Predator stress increase α_1_R sensitivity	Rajbhandari et al., 2015
Prenatal stress decreases α_1_R sensitivity	Systemic/mice	Maternal separation induces α_1_R downregulation	Coccurello et al., 2014
Emotional memory	Amygdala	Chronic α_1B_R activation impaired passive avoidance	Knauber and Müller, 2000
HPA activation	Systemic	α_1_R block inhibits HPA stress response	Yang et al., 2012
Is modulated by chronic stress	Dorsal raphe	Chronic stress impairs α_1_R-induced LTD	Haj-Dahmane and Shen, 2014
**PREFRONTAL CORTEX/EXECUTIVE FUNCTIONS**			
Improves working memory	mPFC	α_1_R activation increases glutamate release	Luo et al., 2015a
Increase working memory	mPFC	α_1_R activation increases mEPSC and response to pressure-applied AMPA and NMDA	Luo et al., 2014b
Improves working memory	PFC	α_1_R activation improves muscimol-induced deficit in working memory	Hvoslef-Eide et al., 2015
Improves working memory	PFC	α_1_R (and α_2_R) activation induces persistent firing	Zhang Z. et al., 2013
Improves working memory	Systemic	Block of α_1_R receptor disrupts go performance	Bari and Robbins, 2013
Improves working memory	PFC	α_1_R prolong persistent activity (up-states)	Zhang Z. et al., 2013
Modulation of working memory	PFC	α_1_R activation decrease NMDA currents	Liu et al., 2006
Modulation of working memory	mPFC	α_1_R activation increases GABA inhibition	Luo et al., 2015b
**CONTROL OF MOVEMENT**			
Motor control worsening	Basal ganglia	α_1D_R KO has improved motor coordination in rotarod	Mishima et al., 2004
Motor impairment	Nucleus accumbens	α_1_R activation impairs motility	Aono et al., 2015
Decrease cerebellar input/motor fine tuning	Cerebellar Purkinje cells	α_1_R activation decrease EPSC	Lippiello et al., 2015
Increase in locomotor activity	Systemic/overall brain	α_1_R agonists increase locomotor activity	Villégier et al., 2003
Regulation of walking/rearing/grooming	N. Accumbens	α_1_R (but **NOT** βR) are involved in reserpine-induced changes in behavior	Verheij et al., 2015
Decrease motor activity	Systemic	decreased exploratory activity	Knauber and Müller, 2000
**SENSORY MODULATION/PLASTICITY**			
Decreased excitability	Visual cortex	α_1_R activation decrease EPSC frequency, amplitude	Terakado, 2014
Increased excitability	Somatosensory cortex	α_1_R activation increase glutamate-induced firing	Devilbiss and Waterhouse, 2000
Decreased excitability	Auditory cortex	Iontophoretic application of α_1_R agonists decrease firing	Manunta and Edeline, 1997
Decreased excitability	Auditory cortex	α_1_R activation decrease glutamatergic response	Dinh et al., 2009
Increased excitability	Auditory cortex	α_1_R activation decreases GABAergic currents from cortical layer 1	Salgado et al., 2011, 2012a
Induces plasticity	Auditory cortex	Phentolamine blocks auditory cortex plasticity induced by electric/optogenetic LC stimulation	Martins and Froemke, 2015
Decreased excitability	Olfactory bulb	α_1_R activation increases GABAergic currents	Zimnik et al., 2013
Increased excitability	Olfactory bulb	α_1_R activation induces neuronal depolarization	Nai et al., 2009
**MEMORY**			
Memory modulation	Entorhinal cortex	α_1_R activation increases GABA release	Cilz et al., 2014
Increases learning and memory	PFC, hippocampus	α_1A_R stimulation improves cognition and learning capability	Doze et al., 2011
Increases learning and memory	PFC, hippocampus	α_1B_R KO mice have reduced learning capability	Spreng et al., 2001
Increases learning and memory	hippocampus	α_1A_R CAM live longer and have improved memory and learning	Collette et al., 2014
**PATHOLOGY/MODELS**			
Antidepressant effect	PFC	Age-dependent effect of tricyclic drugs on α_1_R expression	Deupree et al., 2007
Antidepressant effect	Cortex, cerebellum	Amytryptiline reduces α_1_R density	Ramakrishna and Subhash, 2012
Antidepressant effect, reverse cognitive impairment on an attention-shift task	PFC	Block of α_1_R by quetiapine	Nikiforuk, 2013
Antidepressant effect	Cortex, hippocampus	Electroconvulsive shock increases α_1_R expression	Nalepa et al., 2002
Contributes to drug addiction	BNST	α_1_R activation	McElligott and Winder, 2008
Induces mGlu insensitivity in depression	PFC rodent	α_1_R reduces GluR1 expression (induces downregulation)	Sekio and Seki, 2015
Drug seeking/mobility	Substantia Nigra	α_1_R activation induces drug seeking and promotes mobility	Goertz et al., 2015

## βRs central modulation

Similar to α_2_Rs and α_1_Rs, the distribution of the various subtypes of βRs in the brain is almost ubiquitous in the mammalian brain (Paschalis et al., 2009; Ursino et al., 2009). βRs are, in fact, expressed in both excitatory and inhibitory cells in the cortex as well as in subcortical nuclei (Cox et al., 2008; Salgado et al., 2011; Liu et al., 2014). Among the latter, the amygdala is endowed with an especially high βRs density (Abraham et al., 2008).

### Alertness, wake, and metabolism

Many functions identified for αRs are also brought about by βRs activation. One of them is wake and alertness (Schmeichel and Berridge, 2013). Especially interesting is the effect of βRs activation on astrocytes (Song et al., 2015; Dienel and Cruz, 2016; Sherpa et al., 2016), which induces a decrease in extracellular brain volume. βR are also neuroprotective (Laureys et al., 2014), and decrease endotoxin-induced toxicity (Jiang et al., 2015), possibly by eliciting process retraction in resting microglia (Gyoneva and Traynelis, 2013), in contrast with the induction of neurite growth in cultured cortical primary neurons (Day et al., 2014).

### Cognition and sensory areas

βRs exert their effects in many sensory areas including the somatosensory cortex (Devilbiss and Waterhouse, 2000), the visual cortex (Terakado, 2014), the auditory cortex (Manunta and Edeline, 2004; Salgado et al., 2011), cochlear nucleus, lateral lemniscus, inferior colliculus (Wanaka et al., 1989), and the olfactory bulb (Shakhawat et al., 2015). Activation of βRs impairs sustained attention (Bari and Robbins, 2013), and increases the power (but not the frequency) of γ-oscillations (Haggerty et al., 2013), apparently without impairing cognitive flexibility (Steenbergen et al., 2015).

Similar to the sensory effects of αRs described in the previous sections, the effects of βRs do not necessarily appear to converge onto an unequivocal single function, representing either genuine differences between sensory areas, or recovery of the excitation/inhibition balance through adjustment of synaptic strength or other cellular mechanisms.

### Limbic and motor function

The body of knowledge concerning the effects of βRs on a variety of limbic functions is remarkably consistent with the hypothesis that a high concentration of tonic NE is critical for eliciting or modulating emotion. Fear memory—for instance—is impaired after administration of βR blockers (Fitzgerald et al., 2015; Zhou et al., 2015), and βR activation interferes with fear extinction induced by novel stimuli (Liu et al., 2015). Interestingly, social stress generates microRNA which decreases fear response acting on βRs (Volk et al., 2014). These data indicate that βRs activation is unequivocally associated with fear and fear memory, most likely because of their high expression in the amygdala. βRs are also causally related to anxiety generation, as suggested by the anxiogenic effect of βR-agonist administration (Hecht et al., 2014), and corroborated by elegant experiments where βRs were activated via optogenetic means (Siuda et al., 2015). Interestingly, βR blockers also reduce the anxiogenic effect of cocaine intake (Wenzel et al., 2014), while βR agonist administration within the pre-Botzinger complex increases spontaneous sigh frequency (Viemari et al., 2013).

Perhaps not surprisingly, βRs are important mediators of stress effects. For instance, restraint stress reduces dopaminergic effects but not after blocking βRs (Chang and Grace, 2013). Along the same line, stress elevates LC release of NE, leading to desensitization of βRs, an effect that—if chronic—may give rise to a depressive behavioral phenotype (Porterfield et al., 2012). Motor function appears to be improved by βR activation. βR agonists increase cerebellar GABA input (Di Mauro et al., 2013), increase excitatory input to Purkinje cells, and decrease the threshold for cerebellar LTD (Lippiello et al., 2015), although worsening spatial orientation performance (Robinson et al., 2015).

### Memory and learning

A wide experimental database supports a positive effect of βRs in learning and memory (Salgado et al., 2016). For instance, βR activation increases long-term potentiation (LTP) in the hippocampus and in the neocortex (Laing and Bashir, 2014; Hansen and Manahan-Vaughan, 2015; O'Dell et al., 2015) and memory retrieval, possibly by shutting down an after hyperpolarization activated (AHP) current (Zhang L. et al., 2013; Zhou et al., 2013), by “unsilencing” of silent synapses (Rozas et al., 2015), but also by inducing hippocampal long term depression (LTD; Goh and Manahan-Vaughan, 2013; Lethbridge et al., 2014).

βRs involvement in long-term synaptic plasticity is further indicated by the increased predisposition to long-term changes in both GABAergic synapses (Inoue et al., 2013) and glutamatergic synapses (Maity et al., 2015) following exposure to βRs or stress, in a βR-dependent manner (Grigoryan and Segal, 2013; Grigoryan et al., 2015).

### Neuropsychiatric disease

The role of βRs in neurologic and psychiatric disease is an example of bell-shaped curve: on one hand, a βR *deficit* is associated with CNS malfunction and impairment, on the other one, it is a βRs *hyperactivation* causes distress and neurodegeneration. The former instance is epitomized by the βRs deficits associated with decreased LC-adrenergic function in aging (Santulli and Iaccarino, 2013). The finding of antibodies against βRs in the plasma of chronic fatigue syndrome (Loebel et al., 2016), and the improvement in memory (Dang et al., 2014) and cognitive performance (Phillips et al., 2016) in Down syndrome patients treated with βR agonist highlight the global importance of the βR-dependent component of noradrenergic transmission. The involvement of βRs in Alzheimer disease (AD) symptomatology is somehow controversial. For instance, βR activation appears to increase *tau*-protein phosphorylation—one of the hallmarks of AD (Wang et al., 2013), while routine presentation of novel stimuli is reported to protect from the toxicity from β-amyloid—another important AD marker—through βR activation (Li et al., 2013).

A βR-dependent increase in excitatory transmission in the *bed nucleus stria terminalis* (BNST) has been interpreted as distress factor in drug addiction seeking behavior (Egli et al., 2005), while βR block has been proposed as treatment for depression-related allodynia (Barrot et al., 2009). A possible general interpretation of the body of work related to the function of βRs in the context of stress is that short-term, acute, activation of βRs promotes demanding performances, whereas their chronic stimulation may lead to detrimental consequences of the same functions promoted by βRs short-term action. Table 3 summarizes recent experimental work on βR central function.

**Table 3:** **Central effects of β adrenergic receptors**.

**Measured or putative function of βR activation**	**Brain area**	**Cellular or synaptic effect**	**References**
**ALERTNESS/SLEEP + WAKE TRANSITION/METABOLISM**			
Increase alertness, sensory processing, cognition, memory	Overall	βR activation is necessary for astrocyte aerobic glycolysis	Dienel and Cruz, 2016
Wake promoting	Overall	βR activation decrease extracellular volume	Sherpa et al., 2016
Wake promoting	Preoptic area hypothalamus medial septum	βR (and α_1_R) activation promotes wake	Schmeichel and Berridge, 2013
Wake promoting	Overall brain	βR activation increases astrocyte volume	Song et al., 2015
Decrease neuro-Inflammation	Cortex, hippocampus	βR activation suppress brain inflammation	Ryan et al., 2013
Modulates neuro-inflammation	Cortex	βR activation induces process retraction in resting microglia	Gyoneva and Traynelis, 2013
Induce neuroprotection	Overall brain	βR activation induces neuro-protection	Laureys et al., 2014
Protection from toxicity	Overall	βR activation decrease LPS-induced toxicity	Jiang et al., 2015
Induces axonal growth	Cortex	βR agonists activate glia and induce neurite growth	Day et al., 2014
Increase brain inflammation	Systemic	βR activation increase microglia cytokine production	Johnson et al., 2013
**COGNITION**			
Modulates Working memory/error detection/attention	mPFC	βR are present in mPFC GABAergic interneurons	Liu et al., 2014
Modulates cognition	Hippocampus	βR activation increase power (but not frequency) of gamma oscillations	Haggerty et al., 2013
Weakens working memory/error detection	mPFC	βR activation decreases glutamate release	Luo et al., 2014a
Improves attention	Systemic	Block of βR impairs sustained attention	Bari and Robbins, 2013
Does not affect cognitive flexibility	Systemic cortex, human	Systemic βR block does not affect cognitive flexibility	Steenbergen et al., 2015
**SENSORY ACTIVITY**			
Mixed	Visual cortex	βR activation increases EPSCs	Terakado, 2014
		βR activation increase EPSC amplitude and mIPSC frequency	
Excitation	Auditory cortex	βR agonists facilitate excitatory response	Manunta and Edeline, 2004
Mixed	Auditory cortex	βR agonists facilitate inhibitory response, increase in synchronization	Salgado et al., 2011
Inactivation	Somato-sensory cortex	βR activation decrease glutamate-induced firing	Devilbiss and Waterhouse, 2000
Slow down odor discrimination	Olfactory bulb	βR (and αR) blockage slowed odor discrimination	Shakhawat et al., 2015
**EMOTION/ANXIETY/FEAR/FIGHT-OR-FLIGHT RESPONSE/STRESS**			
Emotional memory	Amygdala	βR block decreases fear memory	Zhou et al., 2015
Decrease discrimination memory	Cortex/amygdala	βR block decrease high arousal induced discrimination memory	Conversi et al., 2014
Promote fear extinction	Amygdala	βR block worsens increase in fear extinction promoted by novel stimuli	Liu et al., 2015
Increase fear response	Amygdala	Interference microRNA generated by social chronic stress decrease fear response by decreasing βR activity	Volk et al., 2014
Fear conditioning	mPFC, amygdala	βR mediated PFC activity increase or decrease induced by fear conditioning	Fitzgerald et al., 2015
Induce anxiety	Amygdala	Peripheral βR activation increases anxiety	Leo et al., 2015
Cognitive effects/Induce anxiety	Cortex/amygdala	βR block improves cognition by blocking anxiety	Hecht et al., 2014
Induces anxiety	Amygdala	Activation of βRs with optogenetics induces anxiety	Siuda et al., 2015
Induce anxiety	Amygdala or BNST to VTA	βR block decreases anxiogenic effects of cocaine	Wenzel et al., 2014
Sighing frequency increase	Pre-botzinger complex brainstem	βR activation increases sigh frequency	Viemari et al., 2013
Stress adaptation	Amygdala	Restraint stress induces dopamine receptor downregulation through βRs	Chang and Grace, 2013
Stress sensitization	PFC, amygdala, hypothalamus	Stress increases NE turnover, desensitization of βR	Porterfield et al., 2012
Long-term changes	Overall	Acute stress induces gene and HPA axis activation	Roszkowski et al., 2016
**MOVEMENT CONTROL/SPATIAL MEMORY**			
Improves spatial orientation	Hippocampus	βR block worsens performance	Robinson et al., 2015
Improves fine tuning of motor control	Cerebellum	βR increases GABA response	Di Mauro et al., 2013
Increases cerebellar function	Cerebellar Purkinje cells	β activation increases EPSCs amplitude and lower LTP threshold	Lippiello et al., 2015
**MEMORY AND LEARNING**			
Increase Memory	PFC	βR activation increases LTP amplitude	Zhou et al., 2013
Memory retrieval	Hippocampus	βR activation decreases sAHP and increases memory retrieval	Zhang L. et al., 2013
Induce memory	Hippocampus	βR activation increases AMPARs insertion (unsilencing of silent synapses)	Rozas et al., 2015
Induce memory/Epigenetic changes	Overall	βR activation triggers epigenetic changes	Maity et al., 2016
Induce memory	DG hippoc	βR activation induces LTP	Hansen and Manahan-Vaughan, 2015
Induce memory	Hippocampus	βR activation increase metaplasticity of glutamatergic synapses	Maity et al., 2015
Induce memory	Hippocampus	βR activation increases LTP	O'Dell et al., 2015
Induce memory	Perirhinal cortex (medial temp lobe)	βR activation induces LTP from amygdala fibers but not within perirhinal cortex	Laing and Bashir, 2014
Induce episodic memory	Dentate Gyrus hippocampus	βR activation induces LTD	Lethbridge et al., 2014
Induce memory	Hippocampus CA1	βR activation induces LTD	Goh and Manahan-Vaughan, 2013
Induce memory	Cortical synaptosomes	βrR activation increase glutamate release	Ferrero et al., 2013
Induce memory	Hippocampus	Prenatal stress decrease βR induction of LTP	Grigoryan and Segal, 2013
Induces Long-term changes in inhibitory circuits	PVN hypothalamus	βR activation induces metaplasticity at GABA synapses	Inoue et al., 2013
Early stress lower threshold for βRs LTP modulation	Hippocampus	Juvenile stress increase LTP sensitivity to βRs	Grigoryan et al., 2015
**βRs IN NEUROPSYCHATRIC PATHOLOGY**			
Aging	LC	Aging correlates with decrement in LC activity	Santulli and Iaccarino, 2013
Occurrence of chronic fatigue syndrome	Whole brain	Antibodies against βRs are evelated in Chronic Fatigue Syndrome	Loebel et al., 2016
Clinical improvement	LC	βR activation increases performance in Down syndrome	Phillips et al., 2016
Memory/Down syndrome	Hippocampus human down syndrome	βR activation improves memory in Down syndrome	Dang et al., 2014
Alzheimer prevention	Hippoc	Novelty activates βRs which protect from amyloid oligomer toxicity	Li et al., 2013
Induction of Alzheimer symptoms	Cortex, hippocampus	βR activation increase tau phosphorylation	Wang et al., 2013
Improve post-traumatic brain injury	Systemic	βR block reduces mortality rate	Ko et al., 2016
Distress induction in drug addiction (seeking) behavior. Intra-BNST βR antagonists inhibition of drug seeking behavior	Bed Nucleus Stria Terminalis	βR activation increases excitatory transmission	Egli et al., 2005
Depression treatment and antiallodynic effect	Systemic/clinic	βR block inhibits pain and decreases depression	Barrot et al., 2009

### Use of β-blockers in the treatment of psychiatric disease

The high expression and high functional relevance of βRs in the amygdala and overall in the initiation of stress response would prompt them as target for pharmacological intervention in the treatment of stress-related psychiatric illness. Administration of the βR blockers, indeed, does decrease the behavioral and biochemical effects of social stress (Wohleb et al., 2011), of restraint stress (Tamburella et al., 2010), and shock-probe defensive burying response (Bondi et al., 2007), possibly by inhibiting cytokine release from microglia, among other effects (Wohleb et al., 2011). Promising results in the treatment of acute effects of stress come from the development of the blood-brain permeable β_3_R agonist *amibegron* (Stemmelin et al., 2008). Activation of βRs may be an important therapeutic component of the antidepressant effect of mirtazapine (Rauggi et al., 2005).

An old hypothesis positing that the therapeutic effect of antidepressant was due to downregulation of βRs (as elicited by TCA treatment; Peet and Yates, 1981) has long been discarded (Charney et al., 1986). βRs agonists have been proposed also in the treatment of the memory impairment associated with psychotic schizophrenia, but the detrimental effects of βR-agonists on working memory and general cognitive flexibility, prevent their routine use (Friedman et al., 2004). In spite of a clear involvement of βRs (and CRH receptors) in amygdala activation in the etiology of PTSD, less or no effective has been the use of βR blocker in the long-term treatment of post-traumatic stress disorder (PTSD; Amos et al., 2014) and schizophrenia (Wahlbeck et al., 2000). βR blockers lack of effectiveness may perhaps be explained by the occurrence of βR internalization induced by their persistent activation. βR internalization was one of the first β-arrestin mediated processes to be described (reviewed in DeWire et al., 2007). Neurons have among the highest expression of non-visual β-arrestin in the whole mammalian body (Gainetdinov et al., 2004). Stress activates β-arrestin mediated internalization of βRs as well as internalization of corticotropin-releasing hormone (CRH) type 1 receptors (Hauger et al., 2009). Either process is an important mechanism of neuronal desensitization to stress response. A related third neuronal desensitization process is the G-protein receptor kinase (GRK)—mediated switch of G-protein functioning from its classic pathway (adenylyl cyclase activation through Gs, in the case of βRs) to a ERK-only pathway (Hauger et al., 2009). This process prevents short-term action of βRs (G_s_-induced activation of adenylyl cyclase) but potentially triggers longer-term mechanism like mitogen activated protein kinase/extracellular signal-regulated kinase (MAPK/ERK), synaptogenesis, and, possibly, maladaptive synaptic plasticity.

## LC-CNS interactions

As discussed earlier, the activation of the PFC-LC-PFC axis is critically important in the stress response (Itoi and Sugimoto, 2010). The extent of the involvement of the LC/central adrenergic system in the coordination of organism *sensory input, decision-making*, and *motor execution* suggests that the LC/NE system plays a critical role in the coordination of all stages of the spatio-temporal pattern of brain activation from quiet wake to periods of intense metabolic demand/stress. At the high end of metabolic demand, detrimental consequences of stress-evoked release of NE may derive from multiple factors, including the simultaneous abnormal release of cytokines—particularly interleukin 6 (IL-6; Li et al., 2015)—which by itself may lead to a wide array of psychiatric consequences from depression to psychosis and anxiety disorders (Atzori et al., 2012). Not surprisingly, an increase in the adrenergic (as well as dopaminergic) tone is an essential component of drug-induced “*high*” (Weinshenker and Schroeder, 2007; Sofuoglu and Sewell, 2009; Fitzgerald, 2013), similar to catecholamine hyper-function during psychotic episodes (Fitzgerald, 2014). An altered sensitivity of adrenergic receptors, or their abnormal function, may thus be a factor shared by a variety of stress-related psychiatric diseases, including post-traumatic stress disorder (George et al., 2013), generalized anxiety (Goddard et al., 2010), fibromyalgia (Clauw, 2014), as well as attention-deficit disorder (Chandler, 2015; Sterley et al., 2016).

We speculate that in a similar fashion, other areas showing differential adrenergic modulation are the motor cortex/cerebellum/striatum complex—responsible for commencing, coordinating, and carrying out rehearsed motor routines and impulsive behavior—as well as the heterogeneous group of brain areas labeled as Limbic System, which generate a variety of positive, negative, and mixed emotional states. The presence of strong anatomical projections from corticotropin-releasing hormone (CRH)-producing limbic areas to the LC is consistent with the hypothesis that negative mood may be a strong trigger for LC activity increase, at least in a physiological functioning brain.

**Figure 5:**
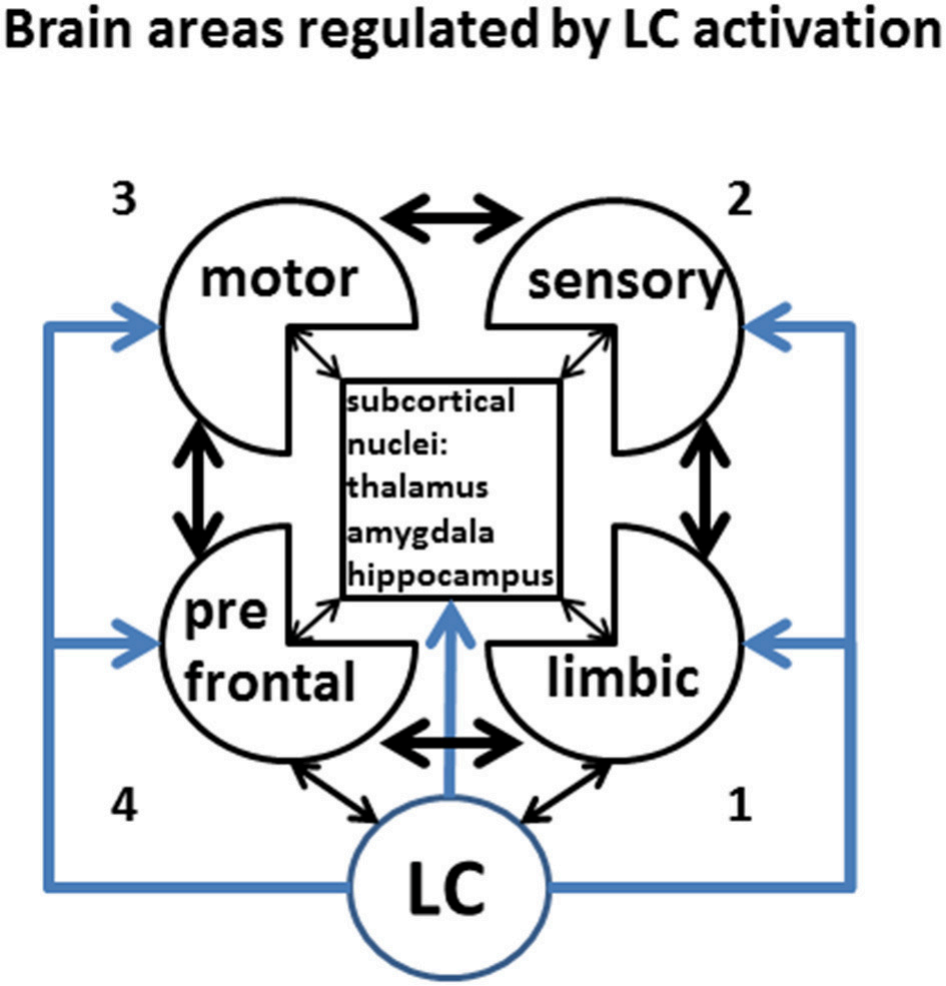
**Brain areas regulated by LC activation**. LC activity controls in a centralized fashion the level of activity and functional connectivity among of virtually all brain area. Keeping in mind that the effects of LC might have regionally specific effects, for the purpose of this discussion we will only consider differential LC effects onto prefrontal, motor, sensory, and limbic cortices, and lump together the activity subcortical nuclei. Different levels of activity are indicated by increasing color intensity, while the strength of inter-regional connectivity will be represented by the thickness of the arrows. This figure represents the legend for the Figures 6–8. The number (1–4) on the side of each sketch represents the putative resting energy demand of each activated state, from the least-demanding (LA) to the most demanding (PFC).

We previously defined as stress any circumstance that raises the energy demand above an expected or biologically bearable threshold. Keeping in mind this idea, the steady-state energetic need of different fully-activated cortical areas varies greatly. For instance, limbic areas appear to be active even during sleep (default network; Buckner et al., 2008), with minimal energy demand. Purely sensory tasks, accompanied by sensory cortex activation, are likely to be the next least energetically demanding areas, as they are endowed with inbuilt circuitry for passive activity during the wake state. On the contrary, effective motor activity requires a combination of intention and sensory-motor coordination, which sets the motor circuitry to a relatively high-energy-demanding position. The highest energy need is requested by the prefrontal cortex, whose “working memory” juggles between multiple tasks including attention, planning future actions based on the retrieval of behavioral rules and sensory information stored earlier, and inhibition of momentary impulse. In addition, a high tone of limbic areas may drive high energy consumption from the PFC, to which is anatomically and functionally bi-directionally connected. It is likely, then, that stress affects to different extents brain areas with different stress-imposed additional energy requirement. Cortical areas in Figure 5 are numbered (1–4) in order of increasing energy need (anticlockwise), starting from limbic areas (1, lower right), sensory areas (2, upper right), motor areas (3, upper left), up to the prefrontal cortex (4, lower left).

### LC-NE induced activation states

**Figure 6:**
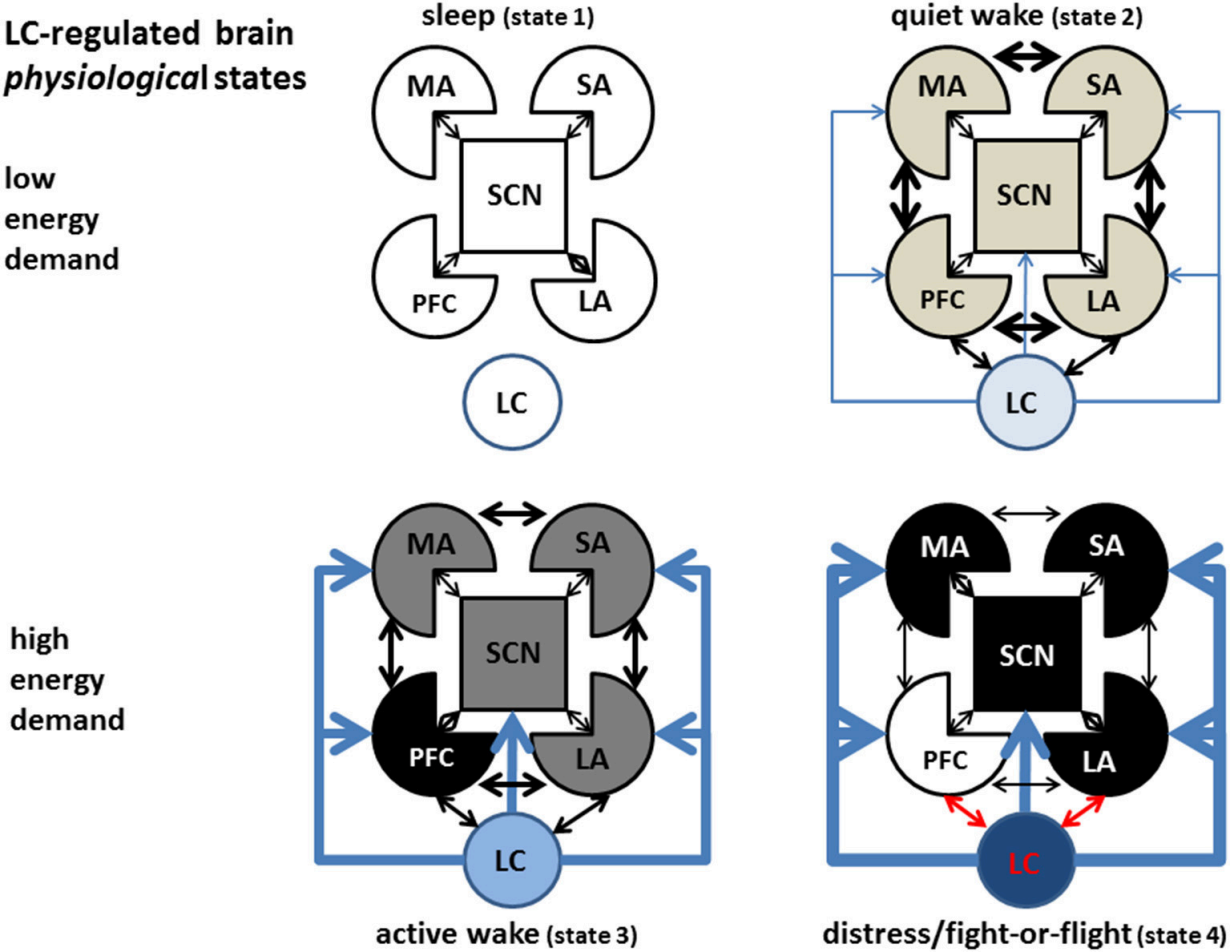
**LC-regulated brain activation states**. Refer to Figure 5 as legend for the representation of different brain areas. While many intermediate states are likely to exist, we depict in the sketch only four of them, in order of energy demand. During the sleep state (upper left) the LC is inactive, all cortices (except possibly limbic cortices) are virtually inactive, maintenance processes (like memory consolidation and basal immune activity) are on-going, while cellular energy content is restored. During quiet wake(upper right), LC is moderately active in the tonic mode, maximizing phasic release of NE which allows optimal intracortical communication and flexible behavioral and decision-making strategies and memory and learning associated with high phasic LC activation and βR-mediated activation. During high-energy demand (stress, lower left), an increased drive in the limbic cortex induces higher LC activation and hyperactivity in other cortical areas relevant to the specific stressor (most often the PFC, but on occasion could be other areas like motor or sensory cortices could be over-activated to carry out specific behavioral tasks). Extreme stress induces hyperactivity in parts of the limbic system, fight- or-flight response (lower right), overdrive and functional shut-down of the PFC, and hyper-activation of motor areas and subcortical nuclei (symbolized by the grid lines, MA: motor areas, SA sensory areas, SCN: subcortical nuclei, PFC: prefrontal cortex, LA: limbic areas, LC: *locus ceruleus*).

Good evidence exists for gap-junction mediated synchronous LC activation (Ishimatsu and Williams, 1996; Rash et al., 2007). Computational modeling supports the hypothesis of simultaneous activation of LC neurons, and simultaneous increase in brain NE (Gao and Holmes, 2007; Patel and Joshi, 2015), although alternative hypothesis have been proposed (Chandler et al., 2014a,b; Chandler, 2015). These observations suggest that different brain states may be elicited by increasing NE concentrations progressively activating ARs from high to low affinity for NE. In each of these states a different combination of tonic and phasic NE levels would give rise to regional differences in the brain activity, as well as to specific patterns of global function. In this section we will describe a largely speculative proposal for a sequence of cortical states in the order of progressively higher energy demand (Constantinople and Bruno, 2011), higher CNS NE levels, and progressive binding to the sequence of adrenoceptors α_2_R, α_1_R, and βRs in the order of affinity from the highest to the lowest (Figure 6). The intensity of gray indicates the level of physiological (non-pathological) activation, the thickness of the arrows indicates the strength of the connectivity between areas.

*Sleep* is the lowest-energy state, corresponding to a virtual absence of adrenergic tone. The whole organism—particularly the CNS—refills metabolic energy stores depleted during the previous wake phases. During this metabolic stage the organism is able to carry out important low-energy demand functions which do not require behavioral performance, like maintenance immune functions, rehearsal of mnemonic segments (Foster and Wilson, 2006) or motor sequence silent replay (Barnes and Wilson, 2014), aimed to synaptic stabilization and episodic or procedural memory consolidation.

The second state in the energy-demand ladder (*quiet wake*) is associated to low LC tonic firing, an active reward system, and mostly positive emotion in limbic areas. In this circumstance, the VTA releases dopamine that stabilizes the motivation axis represented by LC-Nucleus Accumbens-PFC receiving further input from the limbic cortices, and producing an optimal balance in the activity and reciprocal interaction among cortical areas and between cortical and subcortical regions. This state is characterized by optimal and flexible exchange of information between cortical areas and a relatively low physical and mental energetic load. In terms of LC activation/NE release, is associated with low but not nihil LC tonic firing and a high dynamic range for phasic LC responses to novel or salient stimuli, while, in terms of adrenoceptor activation, corresponds to tonic activation of α_2_ adrenoceptors, sporadic activation of α_1_Rs, and memory-promoting activation of βRs during phasic LC activation.

Tonic NE concentrations in the α_1_R activation range would promote a third condition (*active wake*) represented by a series of states characterized by selective activity-dependent enhancement of energy consumption in particular cortical areas. Such areas would be selected depending on the specific demands of the circumstance, driven by a relatively high emotional tone in limbic areas. For instance, during strenuous physical activity, strong sensory engagement, or critical behavioral planning, NE released from LC would selectively depress the activity in non-critical cortical areas in an α_1_R-dependent fashion, through depression of glutamatergic synapses (Dinh et al., 2009; Roychowdhury et al., 2014). At the same time, NE release would enhance energy consumption in critical cortical area(s) (i.e., motor controlling, sensory areas, working memory, or other brain areas) in an activity-dependent fashion, through a combination of phasically activated α_1_- and β-AR activation. This state would be associated with a relatively high tonic level of LC firing, a limited range of phasic LC responses to salient or new stimuli, and a decreased but still functional communication between different cortical areas. In the “*active wake*” state, an in-built circuitry, prepared by evolution for automatic processing, would promote strong but not overwhelming activity in sensory areas, motor areas, as well as in the PFC. The latter would elaborate variable strategies to resolve the specific demands of the contingency for which automatic processing is *not* effective. In social mammals, extinguishing a stressor may require an additional inter-individual interaction (social) component that overburdens the limbic system and is therefore especially vexing on the individual.

At the physiological highest level of energy consumption, the LC would display the strongest *tonic* activation, high tonic NE release, and a limited or inexistent range of *phasic* NE release, corresponding to the “*fight-or-flight*” (FoF) response described in the pioneering work of Cannon reviewed in Fee and Brown (2002), at the extreme of which could lay the *berserk* condition. This state is characterized by strong activation of cortical βARs, strong negative *or* positive emotion, impulsive response, deficient sensory activity, shut-down of the planning (PFC) areas, and scant or inefficient intra-cortical communication (Holmes and Wellman, 2009). This condition would be terminated with either of two outcomes: (1) the elimination of the stressor, resetting of LC activity to low tonic state, reactivation of a temporarily inhibited dopaminergic system, and return of the system to a low-energy state (*sleep* or *idle wake*), or, on the opposite end (2) failure to eliminate the stressor, depletion of organic energy reserve, and, possibly onset of long-term deficit or even death. In humans, this condition may give rise to neuropsychiatric disorder including epilepsy, *burn-out* syndrome, psychosis, depression, or anxiety, depending on the stressor pattern, individual genetic predisposition, and previous life history.

The maximum duration and intensity of high-energy states (*active wake* and *distress/FoF*) bearable by a specific individual would display significant inter-individual differences related to genetics, previous training/experience, and motivational state, and strongly depends on the history of the subject, to the point that periodic and controlled incursions into the stress state may be beneficial to increase the probability of successfully extinguishing unexpected future stressors. The hypothesis is graphically summarized by the sketches in Figures 5, 6.

An oft found bell-shaped dependence of specific PFC-dependent performance on adrenergic activation (Roychowdhury et al., 2012; Sapolsky, 2015) could be interpreted as a transition from a low-energy state (state 2: *quiet wake*), to state 3 (*active wake*), associated with a larger energy mobilization, stronger engagement, and improved cognitive performance (left part of the bell shape curve). At the right end of the curve would lay the transition between state 3 (*stress*) and state 4 (*FoF*, right part of the bell shape), with a massive engagement of βRs in the cortex as well as in subcortical nuclei—particularly the amygdala—and consequent dysfunctional working memory, in favor of an optimal impulsive, automatic, motor response and full-fledged autonomic sympathetic response (Bouret and Sara, 2005; Hains and Arnsten, 2008; Gamo and Arnsten, 2011). Needless to say, a comprehensive theory of energy mobilization in high-demanding states (*active wake and distress/FoF*) should include the role of other global transmitters, particularly acetylcholine, histamine, and 5HT. Such discussion is left for important future work, and falls outside the scope of this review.

It is tempting to speculate further that the regional pattern of energy consumption in this condition may have changed in the course of mammalian evolution, and possibly along mankind history, such that the effects of stress on *motor* and *sensory* cortices used to be a lot more severe during early history/evolution/developmental stages, compared with the effects on the PFC, while the latter has become (is becoming) the major subject—and potential victim—of stress in modern society, particularly for adolescent and adult humans.

### Clinical consequences of stress-induced maladaptive plasticity

Stress notoriously impairs the dynamic balance between sympathetic and parasympathetic autonomic branches, affecting sleep, digestion, endocrine function, by altering the balance between peripheral parasympathetic and sympathetic tones (Grippo and Johnson, 2009; Silvani et al., 2016). Even more consequential, in the CNS, physiological stress of high intensity and/or prolonged duration may lead to β arrestin-mediated internalization of adrenergic receptors, studied in detail for βRs (Stone and Quartermain, 1999), leading in turn to a de-sensitization of βR-mediated central adrenergic pathways (Fu and Xiang, 2015). A possible consequence of intense or prolonged stress could be a decrease in effectiveness of the LC adrenergic system, leading to a decrease in the expression of the β_1_R type (Porterfield et al., 2012) in limbic areas, and to a change in the expression of βR-related effectors in other parts of the limbic system like the hippocampus (Benes et al., 2004).

**Figure 7:**
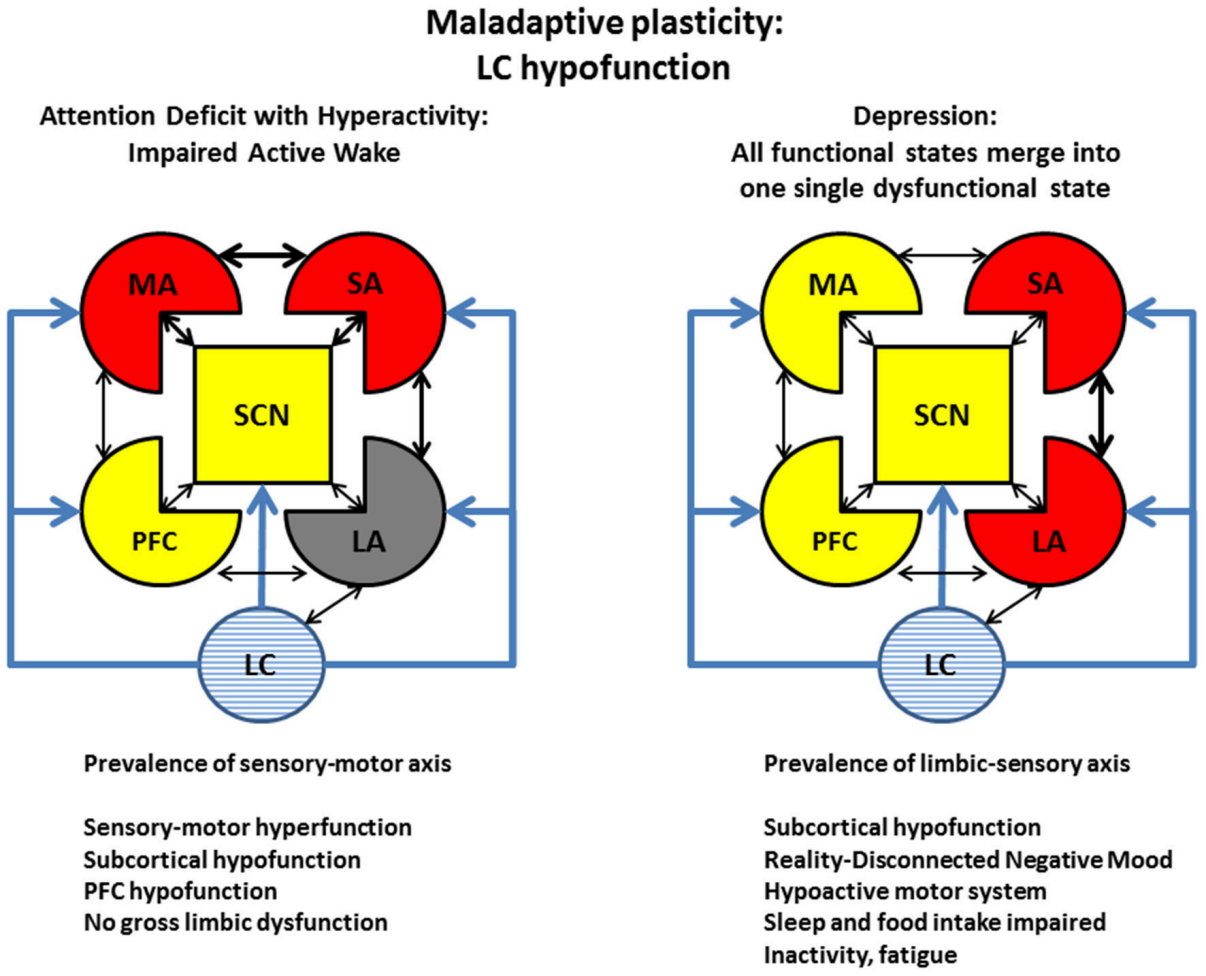
**Maladaptive plasticity: Examples of LC hypofunction**. **Left**: ADHD. Attention deficit disorder with hyperactivity (ADHD) is treated clinically with pro-monoaminergic drugs, particularly with NE re-uptake blockers. This condition may represent a dysfunction of the *active wake* (Figure 6) caused by NE/LC hypofunction. The condition is characterized by a prevalence of a motor-sensory areas and a decrease of working memory and inhibitory control. The deficit should not be considered a severe impairment insofar it is not associated with major alteration of limbic function. **Right**: Depression. The use of NE- (along with 5HT-) reuptake blockers is also in the mainstream treatment for depression. While depressed patients also display similar traits of ADHD subjects, like impaired working memory and low threshold for sensory activation, contrary to ADHD, depression is associated with long-term impairment of limbic function. According to our model, in depression, the normal physiological cycling between the 4 states illustrated in Figure 5 is turned into a single dysfunctional state. Refer to Figure 5 as legend for the representation of different brain areas. Captions as in Figure 6.

**Figure 8:**
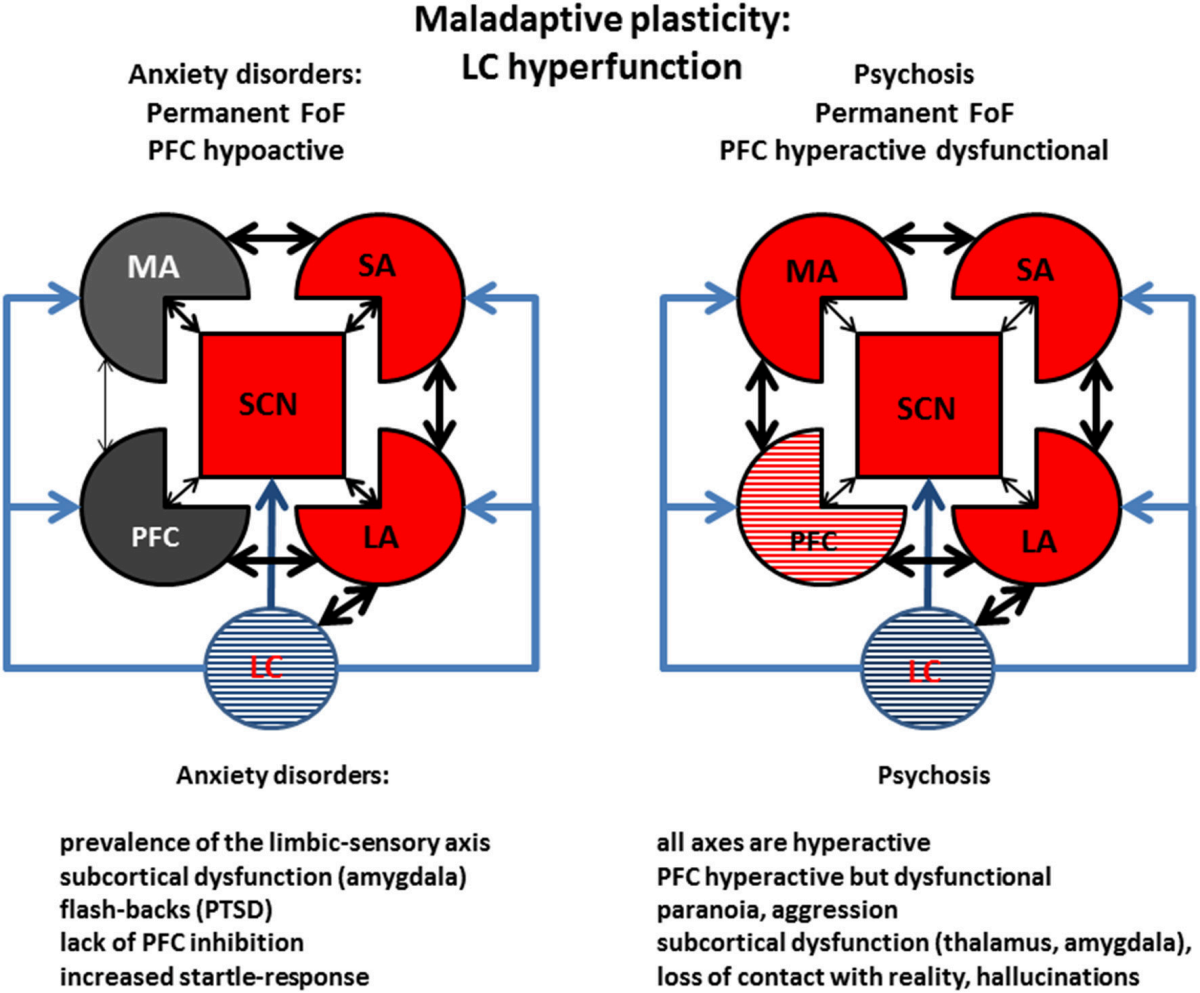
**Maladaptive plasticity: Examples of LC hyperfunction**. **Left**: Anxiety. Prolonged or intense stress may deplete organismic energy stores, possibly along with α_1_R overexpression, and βR β arrestin-induced internalization, leading to sensitization of the limbic areas (limbic cortices and amygdala) and of sensory areas. This condition would simulate a permanent reality-detached state of fight-or-flight. **Right**: Psychosis. Failure to eliminate a stress can turn an anxious condition into psychosis, by furthering the impairment of PFC function, possibly accompanied with aggression. Stress and stimulants may precipitate this condition by increasing monoaminergic—particularly dopaminergic and noradrenergic tone—in the PFC, where catecholamine transporter is responsible for the re-uptake of both molecules. Depression would differ from psychosis mainly in monoaminergic function (decreased in depression but increased in psychosis), causing an exaggerated motor response, but would share with it working memory impairment and sensory and limbic sensitization (compare with Figure 7, captions as in Figure 6).

We represented the two poles of LC/NE function with two examples each, in Figure 7 (LC hypofunction) and Figure 8 (LC hyperfunction). In these figures, red and yellow represent pathologically hyper- or hypo-active areas, respectively. The intensity of the blue stripes inside LC sketch (light in Figure 7 and strong in Figure 8) represents the level of tonic LC activation. As example of LC *hypofunction* we represented attention deficit disorder with hyperactivity (ADHD), in which a monoaminergic hypofunction yields a hypofunctional PFC, which in turn fails to exert a satisfactory inhibitory control on automatic motor activity, associated with sensory distractibility (Figure 7, left). While other monoaminergic deficits (principally dopaminergic) are most likely also involved in ADHD, the efficacy of the NE-reuptake inhibitors like atomoxetine in ADHD treatment corroborates the notion that an LC/NE deficit is a critical component of this condition.

While the causes and mechanisms of clinical depression involve factors other than the LC/NE system, a similar pharmacological argument—the efficacy of selective serotonin/norepinephrine reuptake inhibitors (SNRIs)—also indicates that a deficient noradrenergic system plays a critical role in the treatment of this affliction. In our hypothesis, depression—like ADHD—is also associated with a LC/NE and PFC deficit, but, compared to ADHD, is associated with opposite roles of limbic (hyperactive in depression) and motor (hypoactive in depression) areas (Figure 7, right).

As examples of conditions associated with long-term consequences (maladaptive plasticity) of LC *hyper-function* we selected anxiety disorder (Figure 8, left) and psychosis (Figure 8, right). Anxiety disorders are characterized by hyperactivation of the limbic-sensory axis, with a prevalence of a reality-detached negative mood. Different anxiety disorders may be associated with different degrees of motor activation, ranging from aggression (like in post-traumatic stress disorder), to freezing (like in a rodent response to a predator). Remarkably, anxiety and depression would only differ in terms of motor areas (in)activation, suggesting that further maladaptive plasticity may quickly convert an anxious state into a full-fledged depression, and that the same subject may oscillate between two conditions, which could even take place simultaneously. The large co-morbidity of anxiety and depression (van Tol et al., 2010) corroborates our hypothesis.

An example of even more severe mental condition associated with LC hyperfunction is psychosis (Figure 8, right). This condition is associated with hyperfunction of most cortical areas, promoted by a high monoaminergic tone, leading, in turn, to severe PFC functional impairment. Signs of generalized cortical hyper-function are pathognomonic symptoms of psychosis, like paranoia and hallucinations, as well as aggression. In this condition, prolonged and/or intense stress elicits a type of maladaptive plasticity that sensitizes limbic and sensory areas leading to loss of touch with reality, and—in the most dramatic cases—aggression and gross working memory impairment. Clinical support for a strong involvement of the LC/NE system—together with other monoaminergic systems—in psychoses, is the precipitation of psychotic episodes after intake of drugs including legal or illegal NE and other monoaminergic re-uptake blockers. Up-regulation of α_1_Rs may be a component of PFC impairment observed in the ventral-hippocampal lesion model of schizophrenia (Al-Khairi et al., 2009).

Depression and psychosis share the traits of working memory impairment, some level of detachment from reality, and hypersensitivity of limbic and sensory system, all of which can be triggered by prolonged or intense stress. Clinically, these two conditions may thus represent the result of a parallel process of stress-induced maladaptive plasticity landing on opposite poles of motor drive because of genetics or cultural factor. The similarities between depression and psychosis may explain the presence of both conditions (plus bipolar disease) associated with genes including DISC1 and neuregulins (Blackwood et al., 2007). This hypothesis is further supported by the finding of a reduced monoaminergic drive in a DISC1 animal model displaying depressive symptoms (Lipina et al., 2013).

## Conclusions

Many unanswered questions remain about the role of the LC/NE system. While the presence of gap junctions within the LC has been suggested by anatomical (Rash et al., 2007) and functional (Ishimatsu and Williams, 1996) studies, to our knowledge, synchronous and proportional release of NE in different brain areas following LC activation has not yet been shown unambiguously. The question about simultaneous increase in NE concentration in different brain regions might be answered with precise—in time and space—measurement of monoamine levels, possibly with future developments of already existing electrochemical and microdialysis techniques.

Understanding the nature and extent of the interference between the noradrenergic system and other alertness- and attention-related modulator systems, notably, the serotoninergic, the histaminergic, and the cholinergic systems, and the possible specific role of each neurotransmitter in the global coordination of brain activity is also of critical importance. The presence of reciprocal presynaptic hetero-receptors between neurotransmitters pairs (including GABA and glutamate) may offer important and relatively unexplored mechanism of interaction between different modulatory systems. Hopefully, quantitative modeling will be able to pinpoint a precise correlation between global states induced by NE (and other modulators) and behavioral states.

Computational models reproducing experimental results (Gao and Holmes, 2007; Patel and Joshi, 2015), particularly on the roles of adrenoceptors in behavioral tasks (Chandler et al., 2014b; Chandler, 2015; Somkuwar et al., 2015) are starting to reach a remarkable level of sophistication, and will undoubtedly contribute to integrate the large amount of experimental results collected along many decades on the adrenergic effects on the modulation of intrinsic neuronal conductances and long- and short-term plasticity. Possibly the most important related issue concerns the specific mechanism through which distress elicits maladaptive plasticity, turning a number of physiologically connected, functional, brain areas into a series of dysfunctional circuits as seen in psychiatric disease.

An important and largely overlooked adrenergic mechanisms emerged in the last decade is the role of NE receptors in astrocyte and microglia modulation (O'Donnell et al., 2012; Pankratov and Lalo, 2015). Further, studies will be necessary to integrate the relationships among neuronal function, glial function, and noradrenergic activity.

## Author contributions

MA wrote the article, RC, EE, FG, RS, NS, MM, MT, JP, and HS contributed by designing part of the manuscript structure, with intellectual contributions prior to the manuscript layout, and by writing parts of the manuscript, and revising its full extent. All authors have read, discussed, and accepted the final version of the manuscript.

### Conflict of interest statement

The authors declare that the research was conducted in the absence of any commercial or financial relationships that could be construed as a potential conflict of interest.
